# Autoimmune pancreatitis associated with a pancreatic pseudocyst treated by distal pancreatectomy with splenectomy: case report

**DOI:** 10.1186/1477-7819-12-359

**Published:** 2014-11-27

**Authors:** Xiao-Bo Xu, Ying-Shen Wu, Wei-Lin Wang, Shu-Sen Zheng

**Affiliations:** Division of Hepatobiliary and Pancreatic Surgery, Department of Surgery, First Affiliated Hospital, School of Medicine, Zhejiang University, 79# Qing Chun Road, Hangzhou, 310000 China; Key Laboratory of Combined Multi-organ Transplantation, Ministry of Public Health, 79# Qing Chun Road, Hangzhou, 310000 China; Key Laboratory of Organ Transplantation, Zhejiang Province, 79# Qing Chun Road, Hangzhou, 310000 China

**Keywords:** Autoimmune pancreatitis, Distal pancreatectomy, Pseudocyst

## Abstract

Autoimmune pancreatitis is a unique type of chronic pancreatitis, which is rarely associated with pseudocyst. A 48-year-old lady was admitted to our department with a rapidly growing cystic mass in the pancreatic tail with an elevated concentration of serum carbohydrate antigen 19-9 (CA19-9). She had a history of autoimmune pancreatitis and received steroid treatment. Imaging studies demonstrated a cystic mass in the pancreatic tail. The mass kept growing despite restoration of steroid treatment. Eventually, the patient underwent distal pancreatectomy with splenectomy. Histopathological examination revealed the existence of pseudocyst, significant lymphocytic infiltration, and fibrotic change in the pancreatic tail.

## Background

Autoimmune pancreatitis is a peculiar uniform of chronic pancreatitis. The International Association of Pancreatology presented a set of diagnostic criteria for autoimmune pancreatitis[1]. Like other types of pancreatitis, autoimmune pancreatitis can be complicated with pseudocyst, although this is rarely reported[2–4]. Kawakami *et al*.[2] suggested that corticosteroid treatment should be started immediately when a pseudocyst appears, accompanying autoimmune pancreatitis, because the pseudocyst did disappear with treatment. However, there is no consensus on therapeutic strategy for autoimmune pancreatitis associated with pseudocyst without good response to steroid treatment. Here, we present a patient who was clinically diagnosed with autoimmune pancreatitis with a rapidly growing cystic mass located in the pancreatic tail that was insensitive to steroid therapy. This patient underwent distal pancreatectomy with splenectomy.

## Case presentation

**Figure 1:**
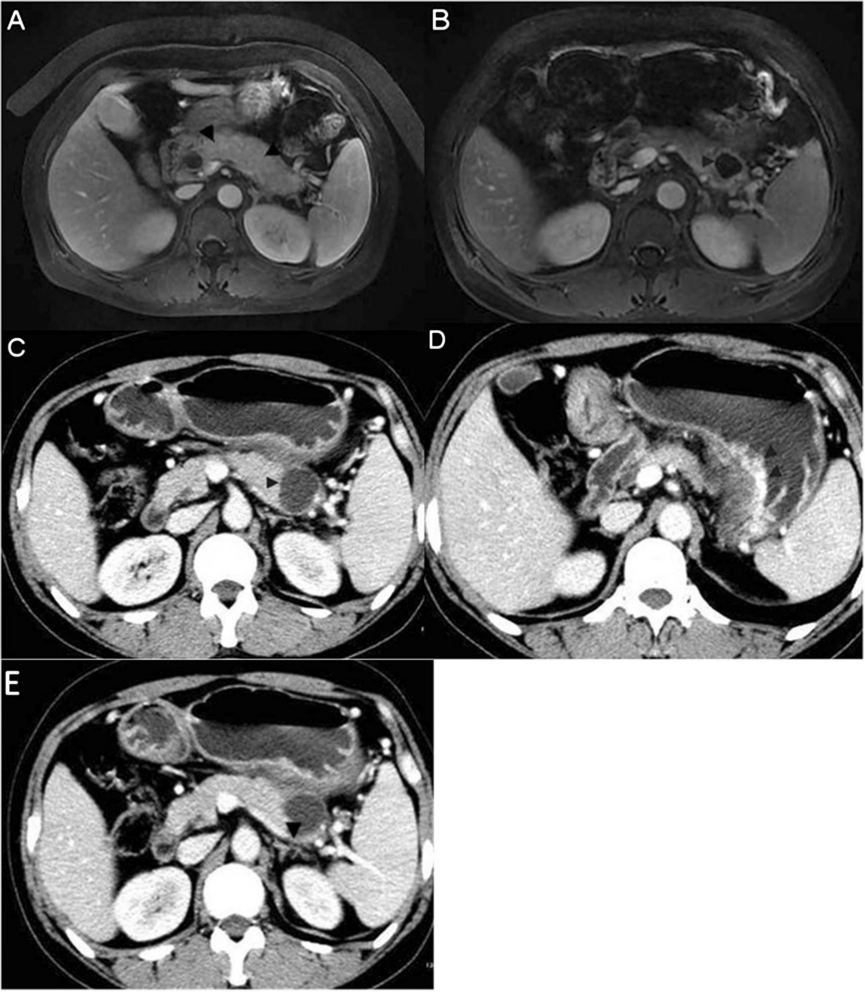
**Diagnostic magnetic resonance imaging (MRI) and computed tomography (CT). (A)** (MRI) diffuse enlargement of pancreas (arrowhead), **(B)** (MRI) cystic mass in tail of pancreas (arrowhead), **(C)** (CT) obviously enlarged cystic mass in tail of pancreas (arrowhead), **(D)** (CT) varices of gastric body (arrowhead), **(E)** (CT) thrombosis in splenic vein (arrowhead).

A 48-year-old lady was referred to our department for a rapidly growing cystic mass in the pancreatic tail with elevated concentrations of serum carbohydrate antigen 19-9 (CA19–9) (71 u/l), which was found during follow-up for autoimmune pancreatitis. She had no history of smoking or drinking. She had initially presented with jaundice 7 months ago. Autoimmune pancreatitis was diagnosed based on her presentation, elevated serum IgG4 (165 mg/dl) level and image findings (Figure 1A). Therefore, prednisone therapy was started at 30 mg/d and tapered by 5 mg every week. The patient’s jaundice gradually vanished. After that, she came to our department periodically for follow-up. A cystic mass of size 3 cm × 2 cm × 2 cm in her pancreatic tail was found 2 months ago, by magnetic resonance imaging (MRI) (Figure 1B). Serum and urine amylase levels were not elevated. The concentration of serum CA19-9 was 53u/l. Taking this information in combination with her history, we diagnosed autoimmune pancreatitis complicated with a pseudocyst. Conservative management was adopted. The patient restored prednisone therapy with 30 mg/d, tapering by 5 mg every week. The patient’s condition was fine without any symptoms or signs. However, 2 months later, she started to feel abdominal distension, especially after meals. Computed tomography (CT) revealed that the cyst had enlarged to 4 cm × 3 cm × 3 cm (Figure 1C), associated with varices in the partial gastric fundus and body in close proximity to the pancreatic tail (Figure 1D) and thrombosis in splenic vein (Figure 1E).

**Figure 2:**
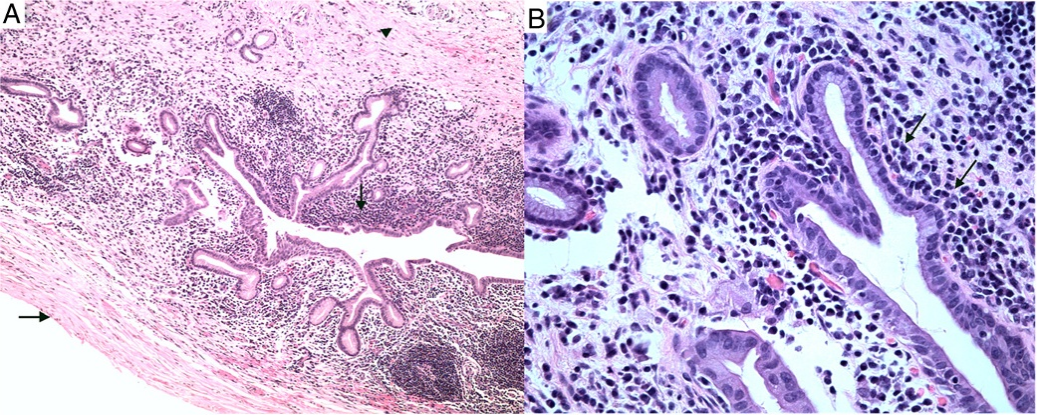
**Histological sections stained by H & E from the resected pancreatic specimen. (A)** Pseudocyst wall without evidence of epithelial lining (horizontal arrow), significant lymphoplasmatic infiltration (vertical arrow) or fibrosis (arrowhead) (original magnification 100×). **(B)** Significant lymphoplasmatic infiltration (arrow) (original magnification 400×).

On hospital admission, the patient continued to take prednisone 5 mg/d. Pertinent laboratory data revealed that serum and urine amylase levels were within normal ranges, as were transaminase and serum bilirubin levels. However, levels of serum CA19-9 (71 u/l) and serum IgG4 (173 mg/dl) were still beyond the upper limit of normal and higher than 2 months previously. Endoscopic retrograde cholangiopancreatography and endoscopic ultrasonography were refused by the patient.Owing to suspicion of cystic neoplasm, drainage of the lesion was not adopted. The patient underwent distal pancreatectomy with splenectomy. The postoperative course was uneventful and the patient was discharged after 7 days. The postoperative serum concentration of CA19-9 decreased to 23 u/l. Histopathological examination revealed a pseudocyst, significant lymphoplasmatic infiltration and fibrosis in the tail of the pancreas (Figure 2), but IgG4 immunostaining was negative.

## Discussion

Cyst formation in autoimmune pancreatitis may be associated with a highly active state of the inflammatory process[3] and severe narrowing of the branched pancreatic ducts[4]. Steroids are thought to induce pseudocyst regression through inhibition of inflammation of the pancreatic duct, thus reducing the stenosis and improving the drainage of pancreatic juice[5]. Hirano *et al*. reported that a growing pancreatic pseudocyst developed in a patient with autoimmune pancreatitis who did not receive corticosteroid treatment[6]. Kawakami *et al*.[2] have advised corticosteroid treatment if a pseudocyst appears, accompanying autoimmune pancreatitis. However, there was no consensus in therapeutic strategy for autoimmune pancreatitis associated with pseudocyst insensitive to steroid treatment. For those cases with ineffective corticosteroid treatment, increasing hormone dosage might be an option, but dosage and duration cannot be determined, and the long-term use of hormone can cause a series of complications.

Operation can be another option in the case of ineffective conservative management. Takita *et al*.[7] presented a patient who was clinically diagnosed with autoimmune pancreatitis accompanied with pseudocyst, was resistant to steroid therapy, and underwent total pancreatectomy with islet autotransplantation. Here, we present the first case of autoimmune pancreatitis with a pancreatic pseudocyst without good response to steroid therapy treated by partial pancreatectomy with splenectomy. In this case, the cystic lesion in the tail of the pancreas was found during follow-up after steroid treatment. The size of the cystic lesion and serum CA19-9 concentration continued to increase despite restoration of steroids. For poor response to steroids of the cystic mass, and the suspicion of cystic neoplasm and thrombosis in the splenic vein, distal pancreatectomy with splenectomy was chosen for this patient.

During postoperative follow-up, serum concentrations of CA19-9 and IgG4 returned to normal, and the patient did not take prednisone or other treatments, but further long-term follow-up is still necessary in case of recurrence of autoimmune pancreatitis or pseudocyst.

## Conclusion

We believe that partial pancreatectomy with splenectomy is another alternative therapy for pseudocyst found in autoimmune pancreatitis when the cyst is insensitive to prednisone treatment, and is accompanied with suspicion of neoplasm and thrombosis in splenic vein.

## Consent

Written informed consent was obtained from the patient for publication of this case report and any accompanying images. A copy of the written consent is available for review by rhe editor-in-chief of this journal.

## Abbreviations

CA19-9: carbohydrate antigen 19-9
CT: computed tomography
H & E: hematoxylin and eosin
MRI: magnetic resonance imaging.
